# Association between legume intake and self-reported diabetes among adult men and women in India

**DOI:** 10.1186/1471-2458-13-706

**Published:** 2013-08-02

**Authors:** Sutapa Agrawal, Shah Ebrahim

**Affiliations:** 1 South Asia Network for Chronic Disease, Public Health Foundation of India, C1/52, First floor, Safdurjung Development Area, New Delhi, India; 2 London School of Hygiene and Tropical Medicine, London, UK

**Keywords:** Legume intake, Diabetes, Men, Women, NFHS-3, India

## Abstract

**Background:**

It is postulated that a diet high in legumes may be beneficial in preventing diabetes. However, little empirical evidence on this association exists in developing countries. We aimed to examine the association between legume intake and self-reported diabetes status in adult men and women in India.

**Methods:**

The analysis is based on a population-based cross sectional study of 99,574 women and 56,742 men aged 20–49 years included in India’s third National Family Health Survey conducted in 2005–06. Association of legume intake, determined by the frequency of consumption of pulses and beans (daily, weekly and occasionally or never), with the reported prevalence of diabetes were estimated using multiple logistic regression after adjusting for frequency of consumption of other food items, BMI status, tobacco smoking, alcohol drinking, watching television, age, education, living standard of the household, residence and geographic regions.

**Results:**

Daily (OR: 0.71; 95% CI: 0.59–0.87; p=0.001) and weekly (OR: 0.66; 95% CI: 0.54–0.80; p<0.001) legumes intake were associated with a significantly reduced prevalence of diabetes among adult Indian women even after controlling for the effects of potentially confounding factors, whereas non-significant inverse associations were observed in men.

**Conclusion:**

Daily or weekly intake of legumes was inversely associated with presence of diabetes in the Indian population. However, this is an observational finding and uncontrolled confounding cannot be excluded as an explanation for the association. More epidemiological research with better measures of legumes intake and clinical measures of diabetes is needed to clarify this relationship.

## Background

The independent association between legume intake and diabetes risk is not well documented, particularly in developing countries. As the prevalence of type 2 diabetes mellitus has been increasing rapidly worldwide, [1] knowledge of risk factors and protective factors associated with diabetes is essential for the development of prevention strategies. Legumes—including pulses, beans, lentils, peanuts, peas, and soybeans—are good sources of fiber and have a low glycemic index (GI) [2]. It has been postulated that a diet high in legumes may be beneficial in preventing diabetes. Consumption of legumes is recommended by the European [3], Canadian [4] and American Diabetes Associations [5] as a means of increasing one’s daily fiber intake and lowering GI for diabetes control. In India, however, diabetes prevalence is increasing in both rural [6,7] and urban [7-10] populations, despite the consumption of traditional diets high in legumes [11].

Epidemiologic studies in the West, where the average daily intake in grams is much lower than in India, have yielded inconsistent associations on legume intake and chronic conditions [2,12-18]. Given the high growing prevalence of diabetes in India, the role of various food items needs to be examined in relation to its prevalence. Legume consumption is ubiquitous in India as more than half the Indian population consumes it daily [19]. There is a dearth of empirical research in India regarding the role of legumes in the prevention of diabetes. India’s third National Family Health Survey (NFHS-3, 2005–06) collected data from 109,041 households on a wide range of dietary, societal, lifestyle, and environmental determinants of morbidity and chronic ailments, including diabetes, for adult men aged 15–54 years and women aged 15–49 years, [19] and covered regions comprising more than 99% of India’s population. These data provide a unique opportunity to study the association between legumes consumption and the prevalence of diabetes. In this paper, we assessed whether daily or weekly intake of legumes was associated with a lower risk of diabetes among adult Indians.

## Methods

### Data

Data from NFHS-3, 2005–06 were used for this study. Details of the survey method including sampling frame and questionnaire used are provided in the basic survey report for all India [19]. Briefly, this survey was designed on the lines of the Demographic and Health Surveys (DHS) (available at http://www.measuredhs.com) that have been conducted in more than 90 developing countries since the 1980s. NFHS has been conducted in India for three successive rounds, each at an interval of five years. NFHS-3 collected demographic, socio-economic, and health information from a nationally representative probability sample of 124,385 women age 15–49 and 74,369 men aged 15–54 residing in 109,041 households. The sample is a multi-stage cluster sample with an overall response rate of 98 percent. All the states of India are represented in the sample (except the small Union Territories), covering more than 99 percent of the country’s population. The analysis in this study is restricted to 99,574 women and 56,742 men aged 20–49 years living in the sample households.

### Response variable

The survey asked several questions relating to the current health status of the respondents, including the question, ‘*Do you currently have diabetes*?’ The survey was conducted using an interviewer-administered questionnaire in the native language of the respondent using a local, commonly understood term for diabetes. A total of 18 languages were used in the survey with back translation into English to ensure accuracy and comparability. No physician diagnosis of diabetes could be obtained to verify self reports and it was not possible to take fasting blood glucose to establish a diagnosis. In our analysis, reported prevalence of diabetes is the outcome of interest.

### Predictor variables and covariates

The survey collected information on demographic and socio-economic factors, anthropometric measurements and dietary intake. Consumption of selected foods was assessed by asking, ‘*How often do you yourself consume the following food items*: *daily, weekly, occasionally or never?*’ This question was asked with respect to the following foods - legumes (including pulses or beans), milk or curd, green leafy vegetables, other vegetables, fruits, eggs, chicken, meat or fish. Frequency of watching television (almost every day, at least once weekly, less than once weekly, not at all) was used as a measure of sedentary behaviour. Use of tobacco was measured as never smoker and ever smoker. Use of alcohol was quantified as drinks almost every day, about once weekly, less than once weekly and never. Indian adult population standard [20] categories of Body Mass Index (BMI, kg/m^2^) were used: ≤18.5 kg/m^2^ (underweight); 18.5 to 22.9 kg/m^2^ (normal), 23.0 to 24.9 kg/m^2^ (overweight), and ≥25.0 kg/m^2^ (obese).

Because the effects of legume intake on the prevalence of diabetes are likely to be confounded with the effects of other factors, it is necessary to statistically control, or adjust for such factors. Control variables were chosen based on earlier evidence, theoretical knowledge, and the availability of the variables in the NFHS-3 data. Earlier studies have associated the prevalence of self-reported chronic conditions with age, sex [25], income, education, religion and rural/urban residence [21-26]. Control variables included in this analysis are: age (20–29, 30–39, 40–49 years); education (illiterate, literate but less than middle school complete, middle school complete but less than high school complete, high school complete or higher); religion (Hindu, Muslim, Christian, Sikh, Other); caste/tribe (scheduled caste, scheduled tribe, other backward class, others, missing caste); wealth status (based on 33 assets and housing characteristics graded lowest, second, middle, fourth, highest) was computed using previously described methods [19]; and place of residence (urban, rural). For a detailed definition of variables see Table 1.

**Table 1:** Sample distribution and prevalence of diabetes (%) among men (n=56,742) and women (n=99,574) aged 20–49 years according to legumes intake and other selected risk factors and background characteristics, India 2005-06

**Characteristics**	**Men**			**Women**		
	**Total number N [%]**	**Who have diabetes N [%]**	**χ**^**2**^p value*	**Total number N [%]**	**Who have diabetes ** **[%]**	**χ**^**2**^p value*
**Legumes intake**			<0.0001			<0.0001
Daily	29863[52.6]	437[1.5]		52440[52.7]	538[1.0]	
Weekly	21705[38.3]	219[1.0]		36597[36.8]	360[1.0]	
Occasionally	4660[8.2]	51[1.1]		9663[9.7]	131[1.4]	
Never	505[0.9]	13[2.6]		852[0.9]	20[2.3]	
**Consumption of other food items**						
*Milk or curd*			<0.0001			<0.0001
Daily	26307[46.4]	391[1.5]		40366[40.5]	492[1.2]	
Weekly	11554[20.4]	117[1.0]		15071[15.1]	138[0.9]	
Occasionally	14757[26.0]	138[0.9]		32918[33.1]	302[0.9]	
Never	4114[7.3]	74[1.8]		11202[11.3]	117[1.0]	
*Green leafy vegetables*			0.149			0.090
Daily	33982[59.9]	453[1.3]		64095[64.4]	674[1.1]	
Weekly	19270[34.0]	231[1.2]		28606[28.7]	286[1.0]	
Occasionally/never	3480[6.1]	35[1.0]		6840[6.9]	89[1.3]	
*Fruits*			<0.0001			<0.0001
Daily	7320[12.9]	125[1.7]		12789[12.9]	206[1.6]	
Weekly	19368[34.1]	255[1.3]		26731[26.9]	276[1.0]	
Occasionally	28484[50.2]	296[1.0]		56336[56.6]	503[0.9]	
Never	1546[2.7]	44[2.8]		3631[3.6]	63[1.7]	
*Eggs*			<0.0001			<0.0001
Daily	2931[5.2]	56[1.9]		3475[3.5]	60[1.7]	
Weekly	20682[36.5]	317[1.5]		28778[28.9]	363[1.3]	
Occasionally	19786[34.9]	201[1.0]		32635[32.8]	287[0.9]	
Never	13330[23.5]	146[1.1]		34647[34.8]	340[1.0]	
Fish intake			<0.0001			<0.0001
Daily	3706[6.5]	90[2.4]		6505[6.5]	149[2.3]	
Weekly	14414[25.4]	238[1.7]		22070[22.2]	304[1.4]	
Occasionally	21818[38.5]	225[1.0]		34242[34.4]	264[0.8]	
Never	16782[29.6]	167[1.0]		36724[36.9]	331[0.9]	
*Chicken or meat*			<0.0001			<0.0001
Daily	706[1.2]	6[0.9]		839[0.8]	14[1.7]	
Weekly	15609[27.5]	269[1.7]		21938[22.0]	292[1.3]	
Occasionally	26135[46.1]	291[1.1]		42222[42.0]	423[1.0]	
Never	14272[25.2]	155[1.1]		34537[34.7]	320[0.9]	
**Body mass index and lifestyle factors**						
*Body mass index (kg/m*^*2*^*)*^*a*^			<0.0001			<0.0001
≤18.5 (Underweight)	15356[28.7]	116[0.8]		41219[43.2]	302[0.7]	
18.5-22.9 (Normal)	26616[49.8]	268[1.0]		30663[32.1]	136[0.4]	
23.0-24.9 (Overweight)	5635[10.5]	128[2.3]		9454[9.9]	153[1.6]	
≥25.0 (Obese)	5881[11.0]	178[3.0]		14169[14.8]	437[3.1]	
*Current tobacco smoking*			0.498			0.514
No	35422[62.4]	450[1.3]		97738[98.2]	1030[1.1]	
Yes	21321[37.6]	270[1.3]		1835[1.8]	19[1.0]	
*Alcohol consumption*			0.362			0.020
Never	35965[63.4]	436[1.2]		97101[97.5]	1037[1.1]	
Occasionally	13054[23.0]	180[1.4]		1067[1.1]	7[0.7]	
Once a week	5676[10.0]	74[1.3]		1010[1.0]	3[0.3]	
Almost everyday	2048[3.6]	31[1.5]		396[0.4]	1[0.3]	
*Frequency of watching TV*			<0.0001			<0.0001
Not at all	10517[18.5]	112[1.1]		35399[35.6]	255[0.7]	
Less than once a week	11420[20.1]	95[0.8]		10438[10.5]	96[0.9]	
At least once a week	9081[16.0]	114[1.3]		10952[11.0]	100[0.9]	
Almost everyday	25717[45.3]	400[1.6]		42763[43.0]	598[1.4]	
**Background factors**						
*Age*			<0.0001			<0.0001
20-29	22842[40.3]	91[0.4]		43196[43.4]	113[0.3]	
30-39	19045[33.6]	179[0.9]		33522[33.7]	342[1.0]	
40-49	14855[26.2]	450[3.0]		22856[23.0]	594[2.6]	
*Education*^b^			<0.0001			<0.0001
Illiterate	11607[20.5]	144[1.2]		45113[45.3]	338[0.7]	
Literate, < middle school	10030[17.7]	111[1.1]		14463[14.5]	192[1.3]	
Middle school completed	26783[47.2]	320[1.2]		31665[31.8]	435[1.4]	
High school complete and above	8311[14.7]	146[1.8]		83284[8.4]	83[1.0]	
*Religion*			0.099			<0.0001
Hindu	46727[82.3]	575[1.2]		80648[81.0]	792[1.0]	
Muslim	6841[12.1]	103[1.5]		12940[13.0]	164[1.3]	
Christian	1290[2.3]	19[1.5]		2526[2.5]	56[2.2]	
Sikhs	1009[1.8]	17[1.7]		1836[1.8]	21[1.1]	
Others^*c*^	876[1.5]	6[0.7]		1624[1.6]	16[1.0]	
*Caste/tribe*^*d*^			<0.0001			<0.0001
Scheduled caste	10670[18.8]	131[1.2]		18260[18.3]	173[0.9]	
Scheduled tribes	4732[8.3]	24[0.5]		8002[8.0]	30[0.4]	
Other backward class	22116[39.0]	256[1.2]		38860[39.0]	368[0.9]	
Others	17414[30.7]	270[1.6]		31440[31.6]	437[1.4]	
Missing caste	1810[3.2]	40[2.2]		3011[3.0]	41[1.4]	
*Wealth index*^e^			<0.0001			<0.0001
Lowest	9103[16.0]	71[0.8]		17286[17.4]	71[0.4]	
Second	10205[18.0]	100[1.0]		18546[18.6]	141[0.8]	
Middle	11533[20.3]	80[0.7]		19698[19.8]	152[0.8]	
Fourth	12634[22.3]	154[1.2]		20925[21.0]	275[1.3]	
Highest	13266[23.4]	316[2.4]		23119[23.2]	411[1.8]	
*Place of residence*			<0.0001			<0.0001
Urban	20779[36.6]	347[1.7]		33355[33.5]	551[1.7]	
Rural	35963[63.4]	373[1.0]		66219[66.5]	498[0.8]	
*Geographic regions*^f^			<0.0001			<0.0001
North	7767[13.7]	60[0.8]		13286[13.3]	116[0.9]	
Northeast	2313[4.1]	23[1.0]		3978[4.0]	28[0.7]	
Central	12971[22.9]	82[0.6]		22250[22.3]	126[0.6]	
East	11810[20.8]	213[1.8]		21913[22.0]	295[1.3]	
West	9279[16.4]	90[1.0]		15052[15.1]	121[0.8]	
South	12603[22.2]	252[2.0]		23096[23.2]	363[1.6]	
Total percent		1.3			1.1	
Number^g^	56742	720		99574	1050	

^a^In NFHS-3, all respondents were weighed using a solar powered scale with an accuracy of ±100 g. Their height was measured using an adjustable wooden measuring board, specifically designed to provide accurate measurements (to the nearest 0.1 cm). Pregnant women and women who had a delivery in the 2 months preceding the survey were excluded from the anthropometric measurements.

^b^Education: illiterate (0 years of education), literate but less than middle school complete (1–5 years of education), middle school complete (6–8 years of education), high school complete or more (9+ years of education).

^c^Others include Buddhist, Jain, Jewish, and Zoroastrian.

^d^Scheduled castes and scheduled tribes are identified by the Government of India as socially and economically backward and needing protection from social injustice and exploitation. Other backward class is a diverse collection of intermediate castes that were considered low in the traditional caste hierarchy but are clearly above scheduled castes. Others is thus a default residual group that enjoys higher status in the caste hierarchy.

^e^The wealth index is based on following assets in the household: household electrification, type of windows, drinking water source, type of toilet facility, type of flooring, material of exterior walls, type of roofing, house ownership, ownership of a bank or post office account, and ownership of a mattress, a pressure cooker, a chair, a cot/bed, a table, an electric fan, a radio/transistor, a black and white television, a colour television, a sewing machine, a mobile telephone, any other telephone, a computer, a refrigerator, a watch or clock, a bicycle, a motorcycle or scooter, an animal-drawn cart, a car, a water pump, a thresher, and a tractor.

^f^Region: North: Delhi, Haryana, Himachal Pradesh, Jammu and Kashmir, Punjab, Rajasthan, Uttaranchal; Northeast: Assam, Arunachal Pradesh, Manipur, Meghalaya, Mizoram, Nagaland, Sikkim, Tripura; Central: Chhattisgarh, Madhya Pradesh, Uttar Pradesh; East: Bihar, Jharkhand, West Bengal, Orissa; West: Maharashtra, Goa, Gujarat; South: Andhra Pradesh, Karnataka, Kerala, Tamil Nadu.

^g^Number of men and women varies slightly for individual variables depending on the number of missing values.

*p-value is the result of a simple Chi-square test for independence. Pearson Chi-square test is used to examine whether association between the dependent variable (self reported diabetes) and predictor variable (such as legume consumption) and other covariates and confounders was statistically significant.

### Statistical analysis

Descriptive statistics were calculated with use of standard methods (such as frequencies and percentages) in men and women separately. Prevalence of diabetes was computed as percentage prevalence. Differences were tested using χ2 tests. Trend tests were also carried out scoring the variables in different categories by using likelihood ratio tests. Multiple logistic regression models were used to estimate the odds ratios of daily and weekly legume intake on risk of diabetes after controlling for potential confounders and also examining the independent effects of risk factors. The following models were constructed to account for potential confounders and mediators: Model 1 presents unadjusted results; Model 2 presents results adjusted for the consumption of other food items and socio- demographic factors which may be confounders; Model 3 presents results adjusted for BMI, lifestyle factors and socio-demographic factors which may be confounders; and Model 4 is adjusted for both confounders and mediators to demonstrate any independent effect of legume intake on diabetes. As certain states and certain categories of respondents were oversampled, in all analyses sample weights were used to restore the representativeness of the sample [19].

As the effects of legume intake on the prevalence of diabetes are likely to vary by sex, due to the large gender differences in nutritional status in India, the susceptibility to disease, and access to treatment and care in a developing country, the analysis was carried out separately for women and men. Results are presented in the form of odds ratios (ORs) with 95 percent confidence intervals (95% CI). The estimation of confidence intervals takes into account the design effects due to clustering at the level of the primary sampling unit. Before carrying out the multivariate models, we tested for the possibility of multicolinearity between the variables. In the correlation matrix, all pair wise Pearson correlation coefficients are <0.5, suggesting that multicolinearity is not a problem. All the analysis including the logistic regression models were conducted using the SPSS statistical software package, version 19.

### Ethical considerations

The NFHS-3 survey received ethical approval from the International Institute for Population Science’s Ethical Review Board. Prior informed consent was obtained from each respondent. The analysis presented in this study is based on secondary analysis of existing survey data with all identifying information removed.

## Results

### Characteristics of the study population and prevalence of diabetes

Table 1 shows the characteristics of the study population segregated by men and women, according to their legumes intake, selected risk factors and socio-economic and demographic characteristics, and the corresponding prevalence of diabetes among them. The overall prevalence of diabetes is higher among men (1.3%) than among women (1.1%). Daily or weekly legume consumption was associated with a lower prevalence of diabetes among men (1.5%) and women (1.0%) than observed in people never eating legumes (men 2.6% and women 2.3%). Diabetes was more common among both men and women who consumed milk or curd, eggs, fish, chicken or meat daily or weekly, never consumed fruits, who were either overweight or obese, who watched television almost every day, and in those who were the oldest age group, lived in urban areas and in wealthier households (all p<0.0001). Strong associations between age and diabetes prevalence were observed. Diabetes prevalence increased according to the wealth of the household and was almost double in urban women and men compared with their rural counterparts. Those living in the southern (men 2.0% and women 1.6%) and eastern regions (men 1.8% and women 1.3%) of India had the highest prevalence of diabetes and those in the central region had the lowest prevalence (men and women both 0.6%). No differences in prevalence were seen for green leafy vegetable consumption or smoking tobacco or alcohol consumption. No clear pattern of prevalence by education was also seen.

### Association between legume consumption, modifiable risk factors and control variables and diabetes risk among men

Unadjusted odds (Model 1, Table 2) of suffering from diabetes are more than 40 percent lower (OR: 0.57; 95% CI: 0.32-0.99) among men who consume legumes daily, 61 percent lower (OR: 0.39; 95% CI: 0.22-0.69) among those who consume legumes at least weekly, 58 percent lower (OR: 0.42; 95% CI: 0.23–0.79) among those who consume them occasionally, as compared to those who never consumed legumes. Controlling for consumption of other food items (in Model 2) retains this inverse association but p value becomes non-significant. The effect of legume intake remains virtually unchanged when BMI and other lifestyle factors are additionally controlled in Model 3. When the socio-economic control variables and other covariates are included (Model 4), the effect of legume intake is not significant although the direction of association remains the same.

**Table 2:** Effect (odds ratios with 95% CI) of legume intake and selected factors on the risk of diabetes among men (n=56,742), India, 2005-06

**Predictors and confounders**	**Model 1 OR [95% CI]**	**Model 2 OR [95% CI]**	**Model 3 OR [95% CI]**	**Model 4 OR [95% CI]**	**p value for trend**
**Legumes intake**					<0.0001
Daily	0.57[0.32–0.99]	0.68[0.38–1.22]	0.62[0.35–1.11]	0.70[0.39–1.26]	
Weekly	0.39[0.22–0.69]	0.56[0.31–1.00]	0.51[0.28–0.91]	0.54[0.30–0.98]	
Occasionally	0.42[0.23–0.79]	0.54[0.29–1.03]	0.52[0.28–0.99]	0.56[0.30–1.07]	
Never^R^	1.00[Reference]	1.00[Reference]	1.00[Reference]	1.00[Reference]	
**Consumption of other food items**					
*Milk or curd*					<0.0001
Daily		0.84[0.63–1.12]		0.79[0.60–1.06]	
Weekly		0.71[0.52–0.98]		0.64[0.47–0.88]	
Occasionally		0.64[0.48–0.87]		0.60[0.44–0.81]	
Never^R^		1.00[Reference]		1.00[Reference]	
*Green leafy vegetables*					0.002
Daily		1.04[0.72–1.49]		1.00[0.69–1.43]	
Weekly		1.17[0.81–1.69]		1.15[0.79–1.66]	
Never/Occasionally		1.00[Reference]		1.00[Reference]	
*Fruits*					<0.0001
Daily		0.34[0.23–0.51]		0.35[0.22–0.50]	
Weekly		0.35[0.24–0.51]		0.38[0.23–0.49]	
Occasionally		0.39[0.28–0.56]		0.43[0.28–0.56]	
Never ^R^		1.00[Reference]		1.00[Reference]	
*Eggs*					0.017
Daily		1.59[1.05–2.39]		1.28[0.84–1.94]	
Weekly		1.34[0.97–1.83]		1.18[0.86–1.62]	
Occasionally		1.09[0.80–1.49]		1.07[0.79–1.47]	
Never^R^		1.00[Reference]		1.00[Reference]	
*Fish*					0.109
Daily		1.52[1.03–2.25]		1.55[1.04–2.33]	
Weekly		1.32[0.94–1.87]		1.32[0.92–1.89]	
Occasionally		1.11[0.82–1.50]		1.14[0.81–1.60]	
Never^R^		1.00[Reference]		1.00[Reference]	
*Chicken or meat*					0.568
Daily		0.35[0.14–0.86]		0.31[0.12–0.82]	
Weekly		0.93[0.63–1.35]		0.88[0.65–1.42]	
Occasionally		0.83[0.58–1.19]		0.72[0.55–1.15]	
Never^R^		1.00[Reference]		1.00[Reference]	
**Body mass index and lifestyle factors**					
*Body mass index (kg/m*^*2*^*)*					<0.0001
≤18.5 (Underweight)			0.93[0.73–1.18]	0.92[0.72–1.16]	
18.5–22.9 (Normal)^R^			1.00[Reference]	1.00[Reference]	
23.0–24.9 (Overweight)			1.67[1.34–2.09]	1.61[1.29–2.00]	
≥25.0 (Obese)			1.84[1.49–2.27]	1.83[1.49–2.27]	
*Current tobacco smoking*					<0.0001
No^R^			1.00[Reference]	1.00[Reference]	
Yes			0.93[0.79–1.10]	0.92[0.78–1.09]	
*Alcohol consumption*					0.016
Never^R^			1.00[Reference]	1.00[Reference]	
Occasionally			1.15[0.95–1.40]	1.15[0.94–1.39]	
Once a week			0.96[0.73–1.26]	0.94[0.71–1.23]	
Almost everyday			0.97[0.66–1.44]	0.97[0.65–1.44]	
*Frequency of watching TV*					<0.0001
Not at all^R^			1.00[Reference]	1.00[Reference]	
Less than once a week			0.86[0.65–1.14]	0.90[0.67–1.19]	
At least once a week			1.23[0.92–1.63]	1.24[0.93–1.65]	
Almost everyday			0.84[0.64–1.10]	0.92[0.70–1.22]	
**Background factors**					
*Age*					<0.0001
20–29^R^				1.00[Reference]	
30–39				2.12[1.62–2.76]	
40–49				6.96[5.46–8.87]	
*Education*					<0.0001
Illiterate^R^				1.00[Reference]	
Literate, <middle school				0.78[0.60–1.01]	
Middle school completed				0.75[0.57–0.94]	
High school complete and above				0.72[0.53–0.99]	
*Religion*					0.092
Hindu^R^				1.00[Reference]	
Muslim				1.22[0.94–1.58]	
Christian				0.67[0.41–1.09]	
Sikhs				1.63[0.92–2.89]	
Others				0.51[0.20–1.28]	
*Caste/tribe*					0.001
Scheduled caste^R^				1.00[Reference]	
Scheduled tribes				0.49[0.29–0.70]	
Other backward class				0.71[0.62–0.97]	
Others				0.78[0.62–0.99]	
Missing caste				1.15[0.84–1.94]	
*Wealth index*					<0.0001
Lowest^R^				1.00[Reference]	
Second				1.36[0.99–1.88]	
Middle				0.92[0.64–1.32]	
Fourth				1.74[1.21–2.49]	
Highest				3.48[2.33–5.19]	
*Place of residence*					<0.0001
Urban				1.07[0.89–1.29]	
Rural^R^				1.00[Reference]	
*Geographic regions*					<0.0001
North^R^				1.00[Reference]	
Northeast				2.02[1.14–3.58]	
Central				1.44[0.98–2.13]	
East				3.06[2.09–4.48]	
West				1.49[1.01–2.20]	
South				3.27[2.27–4.71]	
Number of cases	56,729	56,665	52,746	52,693	

For variable definition see Table 1; ^R^Reference category; Model 1 unadjusted; Model 2 adjusted for consumption of other food items and background factors; Model 3 adjusted for BMI and other lifestyle indicators and background factors; Model 4 adjusted for all.

p-Likelihood ratio test for no difference between the three groups for age and standard of living index ignoring the correlated data. As the urban group was expected to have the highest and the rural group the lowest levels of risk factors and disease,.

trend tests were carried out scoring the groups 1 to 3 and using likelihood ratio tests.

### Association between legume consumption, modifiable risk factors and control variables and diabetes risk among women

Unadjusted odds (Model 1, Table 3) of suffering from diabetes are 57% (OR: 0.43; 95% CI: 0.27-0.67), 59% (OR: 0.41; 95% CI: 0.26-0.65) and 43% (OR: 0.57; 95% CI: 0.36-0.92) lower among those who consumed legumes daily, weekly or at least occasionally, respectively, compared to those who never consumed legumes. Controlling for consumption of other food items (in Model 2) maintains this significant inverse relationship. The effect of legumes intake remains virtually unchanged (OR ranges from 0.46 to 0.71) when BMI and other lifestyle factors are additionally controlled in Model 3. Even when the socio-economic control variables are included in Model 4, effect of daily (OR: 0.55; 95% CI: 0.34-0.88) or weekly (OR: 0.56; 95% CI: 0.35-0.90) legume consumption still has a reduced and statistically significant effect on the prevalence of diabetes among women.

**Table 3:** Effect (odds ratios with 95% CI) of legumes intake and selected factors on the risk of diabetes among women (n=99,574), India, 2005-2007

**Predictors and confounders**	**Model 1 OR [95% CI]**	**Model 2 OR [95% CI]**	**Model 3 OR [95% CI]**	**Model 4 OR [95% CI]**	**p value for trend**
**Legumes intake**					0.409
Daily	0.43[0.27–0.67]	0.53[0.33–0.85]	0.46[0.29–0.74]	0.55[0.34–0.88]	
Weekly	0.41[0.26–0.65]	0.56[0.35–0.89]	0.51[0.32–0.82]	0.56[0.35–0.90]	
Occasionally	0.57[0.36–0.92]	0.71[0.44–1.16]	0.71[0.43–1.15]	0.74[0.45–1.20]	
Never^R^	1.00[Reference]	1.00[Reference]	1.00[Reference]	1.00[Reference]	
**Consumption of other food items**					
*Milk or curd*					0.005
Daily		1.09[0.87–1.37]		1.05[0.83–1.32]	
Weekly		0.99[0.76–1.29]		1.00[0.77–1.30]	
Occasionally		1.02[0.81–1.27]		1.01[0.81–1.27]	
Never^R^		1.00[Reference]		1.00[Reference]	
*Green leafy vegetables*					0.742
Daily		0.90[0.70–1.15]		0.88[0.69–1.14]	
Weekly		0.99[0.77–1.28]		1.00[0.77–1.29]	
Never/occasionally^R^		1.00[Reference]		1.00[Reference]	
*Fruits*					<0.0001
Daily		0.45[0.32–0.62]		0.44[0.32–0.61]	
Weekly		0.37[0.27–0.50]		0.36[0.27–0.49]	
Occasionally		0.45[0.34–0.60]		0.46[0.34–0.61]	
Never^R^		1.00[Reference]		1.00[Reference]	
*Eggs*					0.106
Daily		1.06[0.75–1.50]		0.99[0.69–1.41]	
Weekly		0.97[0.76–1.23]		0.97[0.76–1.25]	
Occasionally		0.93[0.72–1.16]		0.96[0.75–1.22]	
Never^R^		1.00[Reference]		1.00[Reference]	
*Fish*					0.225
Daily		1.25[0.91–1.70]		1.15[0.84–1.58]	
Weekly		1.12[0.84–1.48]		1.05[0.79–1.40]	
Occasionally		0.83[0.63–1.08]		0.81[0.61–1.07]	
Never^R^		1.00[Reference]		1.00[Reference]	
*Chicken or meat*					0.267
Daily		1.18[0.64–2.17]		1.05[0.55–1.98]	
Weekly		1.02[0.76–1.38]		1.02[0.77–1.38]	
Occasionally		1.12[0.84–1.47]		1.11[0.84–1.48]	
Never^R^		1.00[Reference]		1.00[Reference]	
**Body mass index and lifestyle factors**					
*Body mass index (kg/m*^*2*^*)*					<0.0001
≤18.5 (Underweight)			0.78[0.63–0.97]	0.79[0.63–0.98]	
18.5–22.9 (Normal)^R^			1.00[Reference]	1.00[Reference]	
23.0–24.9 (Overweight)			1.69[1.39–2.07]	1.67[1.37–2.04]	
≥25.0 (Obese)			2.52[2.13–2.95]	2.50[2.13–2.94]	
*Current Tobacco smoking*					0.703
No^R^			1.00[Reference]	1.00[Reference]	
Yes			1.26[0.79–2.02]	1.24[0.77–2.00]	
*Alcohol consumption*					0.015
Never^R^			1.00[Reference]	1.00[Reference]	
Occasionally			0.85[0.40–1.80]	0.86[0.40–1.83]	
Once a week			0.46[0.14–1.53]	0.51[0.15–1.68]	
Almost everyday			0.53[0.10–2.84]	0.63[0.12–3.37]	
*Frequency of watching TV*					<0.0001
Not at all^R^			1.00[Reference]	1.00[Reference]	
Less than once a week			0.97[0.74–1.21]	0.96[0.75–1.22]	
At least once a week			0.81[0.62–1.03]	0.80[0.62–1.03]	
Almost everyday			0.88[0.76–1.12]	0.91[0.75–1.11]	
**Background factors**					
*Age*					<0.0001
20–29^R^				1.00[Reference]	
30–39				3.29[2.64–4.09]	
40–49				7.82[6.32–9.68]	
*Education*					<0.0001
Illiterate^R^				1.00[Reference]	
Literate, < middle school				1.38[1.14–1.68]	
Middle school completed				1.49[1.24–1.79]	
High school complete and above				0.99[0.73–1.34]	
*Religion*					0.215
Hindu^R^				1.00[Reference]	
Muslim				1.23[1.01–1.51]	
Christian				1.58[1.17–2.14]	
Sikhs				0.97[0.61–1.56]	
Others				1.12[0.66–1.92]	
*Caste/tribe*					0.125
Scheduled caste^R^				1.00[Reference]	
Scheduled tribes				0.56[0.37–0.86]	
Other backward class				0.87[0.71–1.05]	
Others				0.97[0.79–1.18]	
Missing caste				0.85[0.58–1.25]	
*Wealth index*					<0.0001
Lowest^R^				1.00[Reference]	
Second				1.64[1.22–2.21]	
Middle				1.41[1.03–1.92]	
Fourth				1.87[1.36–2.57]	
Highest				1.93[1.35–2.74]	
*Place of residence*					<0.0001
Urban				1.45[1.25–1.69]	
Rural^R^				1.00[Reference]	
*Geographic regions*					<0.0001
North^R^				1.00[Reference]	
Northeast				1.08[0.67–1.73]	
Central				1.05[0.80–1.39]	
East				2.21[1.67–2.91]	
West				0.97[0.73–1.29]	
South				1.71[1.31–2.24]	
Number of cases	100,380	100,224	95,831	95,706	

For variable definition see Table 1; ^R^Reference category; Model 1 unadjusted; Model 2 adjusted for consumption of other food items and background characteristics; Model 3 adjusted for BMI and other lifestyle indicators and background characteristics; Model 4 adjusted for all.

## Discussion

Legume consumption is nearly ubiquitous in Indian diets – more than 99% of the study population reported consuming some pulses or beans preparation either daily, weekly or occasionally. Overall, we found daily or weekly legumes intake were associated with a significantly reduced prevalence of diabetes among adult Indian women whereas non-significant inverse associations were observed in case of men. The association is robust after controlling for other risk factors such as consumption of other food items, BMI, tobacco smoking, alcohol drinking, and a range of socio-economic and demographic characteristics.

Our study is the first cross sectional, population-based study to look at frequency of legume consumption and prevalence of diabetes in India, and adds to the limited data on the associations between legume intake and diabetes prevalence in developing countries. The results of this study are in line with other epidemiologic studies focusing on legumes specifically, which show inverse associations between legumes and diabetes in some of the studies [12,13,17], but not in all [14-16,18]. However, in studies conducted in Asian countries, the inverse associations are primarily due to soy intake [2,17,27]. Evaluations of dietary patterns have identified legumes as an important component of both the ‘prudent diet’ [28] and ‘Mediterranean diet’ [29], which have been associated with a lower risk of diabetes in some [30,31] but not all large cohort studies [28].

It has been suggested that diets high in legumes are beneficial in preventing and managing diabetes, as they are whole grain foods with high insoluble fibre and low glycemic index [32]. The protective effect of legumes on diabetes may be due to multiple biological reasons, including increased fiber content in the diet [33], a reduction in the glycemic index of mixed meals [34], or both. In addition, legumes contain polyphenols, such as isoflavones and lignans, which have an antioxidant effect and may be responsible for the protective role of legumes against the development of diabetes [2]. Though there are several plausible mechanisms by which legumes could reduce diabetes risk and improve glycemic control, some uncertainties still remain [35]. It is possible that this protection is afforded by the intact structure of the pulses slowing digestion and partially restricting absorption of the glycemic carbohydrate [35].

The prevalence of self-reported diabetes in this large nationally representative survey was comparatively low (about 1%) reflecting the young age of this population and the use of self-reports rather than biochemical assessments. Estimates from a recent study of rural–urban migrants showed an age-adjusted prevalence of diabetes (diagnosed using both self-reports and fasting blood glucose in relatively affluent populations) of 10–15 percent in urban people and 5–6 percent in rural people of similar age to those recruited in NFHS-3 [36]. In most urban areas of India the health system is sufficiently developed to diagnose symptomatic diabetes, but at younger ages (<30 years) diabetes may not be symptomatic, and thus NFHS-3 prevalence estimates are undoubtedly conservative, particularly for rural India where diagnosis may be much less likely to occur. However, this ascertainment bias is unlikely to have been differential with respect to legume consumption.

It is necessary to acknowledge that our study has several limitations. Misclassification of dietary information, although unavoidable, would most likely not allow for true associations. Again, there is a possibility that the information derived from the NFHS-3 questionnaire, while critical to measure true dietary intake, may not meet the standards of validity [37] despite the fact that NFHS-3 is a part of the Demographic and Health Surveys (http://www.measuredhs.com) conducted in more than 90 countries, and a similar questionnaire seems to get a fairly valid overall picture of frequency of dietary intake in a population. Another limitation of our study is reliance on self-reports of diabetes. This has resulted in a marked underestimation of prevalence, and its focus on people <60 years in whom diabetes is less common [25]. Self-reported data, especially in rural areas, can be flawed owing to several factors such as lack of awareness, low educational status, limited access to health services and hesitation to disclose diagnosed diseases [25]. Moreover, we were also unable to distinguish between Type 1 and 2 diabetes diagnoses. Under and over reporting could lead to a biased estimation of the association between dietary factors and diabetes. Although we adjusted for several confounding variables, we cannot exclude the possibility of residual confounding. However, if this was the case, similar effects would be expected for other dietary components that are also related to greater affluence, which were not seen.

In these analyses, the cross-sectional design precludes causal inferences and we were limited to the questions used to elicit lifestyle and dietary information. Given the high proportion of undiagnosed diabetes in developing countries (http://www.worlddiabetesfoundation.org) where less than half of people with diabetes are diagnosed, there is a possibility that the exposure was associated with the likelihood of testing for diabetes, which may result in detection bias.

Importantly, the entire study was with known diabetic subjects who might have altered their diet and hence increased or decreased legume consumption due to dietary advice based on diabetes control and on the complications of diabetes like nephropathy. General dietary advice given to diabetic subjects is to include more whole grains and legumes, as evident in the results shown in Table 1, where more than 90 percent of the self-reported diabetics did report ‘daily’ or ‘weekly’ consumption of legumes. Other foods reported also reveal this fact - eggs and fruits ‘daily’ reported by fewer diabetic subjects whereas green leafy vegetables ‘daily’ reported by a larger number of diabetics - all suggest that the dietary choices of self-reported diabetic subjects might have been modified to manage diabetes. Despite these shortcomings, rigorous precautions were taken in the NFHS to obtain reliable self-reported data. The survey used the local terminology and commonly understood term of the disease, rigorously trained interviewers and supervisors and instituted standard quality checks.

## Conclusions

In this large, cross sectional, population-based study of the adult Indian population, daily and weekly intake of legumes was associated with a reduced diabetes prevalence among women, whereas a non significant inverse association was found in men. These results add to the evidence that shows the beneficial effect of consuming legumes in countering the development of diabetes. However, the extent to which legumes contribute to the beneficial effect in terms of prevention and management of diabetes remain to be quantified. These findings need further corroboration by longitudinal and clinical studies but may well have public health significance in the Indian population. More epidemiological research with better measures of legumes intake and clinical measures of diabetes are needed to validate the findings in a developing country.

## Competing interest

Both authors declare that they have no competing interests.

## Authors’ contributions

SA conceived the article. SA conducted and SE supervised the statistical analysis. SA wrote the paper and SE revised it for important intellectual content. All authors read and approved the final manuscript.

## Pre-publication history

The pre-publication history for this paper can be accessed here:

http://www.biomedcentral.com/1471-2458/13/706/prepub
